# Randomised trial reports continue to be islands of evidence, with few continents in sight

**DOI:** 10.1186/1745-6215-14-S1-O95

**Published:** 2013-11-29

**Authors:** Michael Clarke, Sally Hopewell

**Affiliations:** 1 All-Ireland Hub for Trials Methodology Research, Belfast, UK; 2 Centre for Statistics in Medicine, Oxford, UK; 3 French Cochrane Centre, INSERM, Paris, France

## Background

Existing evidence should provide ethical, scientific and environmental justification for a new trial and users of randomized trials need to see their findings within the context of similar trials. Since 1997, *Annals of Internal Medicine*, *BMJ*, *JAMA*, *Lancet*, and *New England Journal of Medicine* have been assessed to see if results are placed in context and, more recently, to see if systematic reviews are used to justify the trial.

## Methods

We assessed each May 2012 issue of these journals to identify reports of randomized trials. Introduction sections were categorised as first trial, updated systematic review used in the design, systematic review mentioned, other trials mentioned, and no other trials mentioned with no claim to be the first trial. Discussion sections were categorised as first trial, systematic review integrating the new trial, systematic review mentioned, and no apparent systematic attempt to place findings in full context.

## Results

35 reports of randomized trials were included. Introduction sections: 5 were said to be the first trial, 1 used an updated systematic review in the design, 13 discussed previous systematic reviews, 10 mentioned other trials, and 6 didn't mention other trials or claim to be the first. Discussion sections: 2 were said to be the first trial, 2 contained a systematic review integrating the new trial, 11 mentioned a systematic review, and 20 made no apparent systematic attempt to place findings in full context. There was variability across the journals.

## Conclusions

Many trials still don't use systematic reviews in their design and reporting.

